# Prevalence, risk factors and multilocus genotyping of *Enterocytozoon bieneusi* in farmed foxes (*Vulpes lagopus*), Northern China

**DOI:** 10.1186/s13071-016-1356-1

**Published:** 2016-02-05

**Authors:** Xiao-Xuan Zhang, Wei Cong, Zhi-Long Lou, Jian-Gang Ma, Wen-Bin Zheng, Qiu-Xia Yao, Quan Zhao, Xing-Quan Zhu

**Affiliations:** State Key Laboratory of Veterinary Etiological Biology, Key Laboratory of Veterinary Parasitology of Gansu Province, Lanzhou Veterinary Research Institute, Chinese Academy of Agricultural Sciences, Lanzhou, Gansu Province 730046 PR China; College of Animal Science and Technology, Jilin Agricultural University, Changchun, Jilin Province 130118 PR China; Jiangsu Co-innovation Center for the Prevention and Control of Important Animal Infectious Diseases and Zoonoses, Yangzhou University College of Veterinary Medicine, Yangzhou, Jiangsu Province 225009 PR China

**Keywords:** *Enterocytozoon bieneusi*, Foxes (*Vulpes lagopus*), Prevalence, Genotyping, Northern China

## Abstract

**Background:**

Microsporidiosis is a common disease in animals and humans around the world. *Enterocytozoon bieneusi* is the most common microsporidian species in humans. Many animal species may be a potential source of human microsporidiosis. However, information concerning prevalence and genotypes of *E. bieneusi* infection in farmed foxes (*Vulpes lagopus*) is scarce. Therefore, the present study examined prevalence, risk factors and genotypes of *E. bieneusi* in farmed foxes in northern China using a genetic approach.

**Results:**

Of 302 fecal samples from farmed foxes, 37 (12.25 %, 95 % CI 8.55–15.95) were PCR-positive for *E. bieneusi*, and the prevalence was highly associated with the farming mode in that foxes raised outdoors (26.03 % positive, 95 % CI 18.91–33.15) had a significantly higher *E. bieneusi* prevalence than those raised indoors. Eleven internal transcribed spacer (ITS) genotypes were identified among the positive samples: four known *E. bieneusi* genotypes (Peru 8, Types IV, CHN-DC1 and D) and seven novel genotypes (NCF1-NCF7). Genotype NCF2 was the commonest (*n* = 13) and was found in five farms across three provinces (Jilin, Heilongjiang and Hebei). All genotypes belonged to phylogenetic group 1. Multilocus sequence typing (MLST) analyses revealed additional diversity.

**Conclusions:**

These findings indicate the presence of zoonotic *E. bieneusi* infection in farmed foxes in northern China. This is also the first report of genotypes Peru8, CHN-DC1 and Type IV, and seven novel genotypes (NCF1-NCF7) in farmed foxes by ITS combining with microsatellite and minisatellite markers for the first time. The results will provide baseline data for preventing and controlling *E. bieneusi* infection in farmed foxes, other animals and humans.

## Background

Microsporidia form an important group of obligate intracellular parasites, and the 1300 named species can infect virtually all animals [1–4]. Fourteen species in eight genera have been reported in humans [5]. *Enterocytozoon bieneusi*, *Encephalitozoon cuniculi*, *Encephalitozoon intestinalis*, and *Encephalitozoon hellem* are the major microsporidians infecting humans worldwide [5], with *E. bieneusi* being the most common, responsible for 90 % of all microsporidian infections [6]. The first case of human infection with *E. bieneusi* was reported in an AIDS patient with chronic diarrhea [6, 7]. Since *E. bieneusi* was detected in pig feces, animals have slowly become recognized as the main intermediate hosts of this pathogen [4, 8]. Feces containing *E. bieneusi* spores can contaminate water or food, leading to microsporidiosis outbreaks [9].

The internal transcribed spacer (ITS) region of the ribosomal RNA (rRNA) gene cluster has been widely used to evaluate the zoonotic risk of *E. bieneusi* [10–13]. Thus far, two large groups have been documented by phylogenetic analysis of ITS sequences, namely a zoonotic group (Group 1) which can infect both humans and animals, and a number of host-adapted groups (Groups 2–5 and outlier genotypes in dogs). More than 200 distinct non-human genotypes are known [14–18]. Some additional small groups have also been found in recent years: Group 7 in Nigerian AIDS patients, Group 6 in urban wastewater in China, and Group 8 was also found in some animals in recent studies [19, 20].

China has abundant animal resources, but only limited information is available regarding prevalence and genotypes of *E. bieneusi*. Most studies in China have reported on *E. bieneusi* in pigs, dogs, cats, cattle, sheep, humans, captive snakes, pandas, some nonhuman primates, and environmental samples [13, 14, 17, 21–26]. Foxes have been the subject of only one study, which was conducted in Harbin City [27]. To improve the information of the distribution of *E. bieneusi* genotypes and estimate the zoonotic potential of *E. bieneusi* infection in foxes, a cross-sectional study of 302 farmed foxes (*Vulpes lagopus*) in northern China was conducted.

## Methods

### Specimen collection and DNA extraction

#### Ethical statement

This study was approved by the Animal Ethics Committee of Lanzhou Veterinary Research Institute, Chinese Academy of Agricultural Sciences (Approval No. LVRIAEC2014-011). In total, 302 fecal samples were randomly collected from healthy farmed foxes from Jilin province (3 farms, 91 foxes), Heilongjiang province (2 farms, 70 foxes) and Hebei province (3 farms, 141 foxes), northern China in 2014. Each fecal sample was collected using sterile gloves immediately after the animal had defecated, and was then stored on ice. In the laboratory, genomic DNA was immediately extracted from feces using an EZNAR Stool DNA kit (OMEGA, USA) according to the manufacturer’s instructions and stored at −20 °C until required for PCR analyses. Information regarding geographical origin, farming mode, gender and age of source fox were acquired for each fecal sample.

### PCR amplification

The genotypes/subtypes of *E. bieneusi* in farmed foxes in northern China were determined using nested PCRs with primers listed in Table 1 [13]. Each reaction (25 μl) contained 1 x Ex *Taq* buffer (Mg^2+^ free), 2 mM MgCl_2_, 200 μM each deoxy-ribonucleoside triphosphate (dNTP), 0.4 μM of each primer, 0.625 U of Ex *Taq* DNA polymerase (TAKARA, Japan), and 2 μl of DNA template. Cycling conditions consisted of preheating at 94 °C for 5 min, then 35 cycles of 94 °C for 45 s, annealing at 55–58 °C (Table 1) for 45 s, and 72 °C for 1 min, followed by a final extension at 72 °C for 10 min. Positive and negative controls were included in each test. PCR products were visualized under UV light after electrophoresis in 2 % agarose gels containing GoldView™ (Solarbio, China).

**Table 1:** Primers used in the study, annealing temperatures used in the PCRs and expected sizes of the PCR products

Gene	Primer	Sequence (5′-3′)	Annealing temperature (°C)	Fragment length (bp)	References
ITS	F1	GGTCATAGGGATGAAGAG	55	392	13
	R1	TTCGAGTTCTTTCGCGCTC			
	F2	GCTCTGAATATCTATGGCT	55		
	R2	ATCGCCGACGGATCCAAGTG			
MS1	F1	CAAGTTGCAAGTTCAGTGTTTGAA	58	675	13
	R1	GATGAATATGCATCCATTGATGTT			
	F2	TTGTAAATCGACCAAATGTGCTAT	58		
	R2	GGACATAAACCACTAATTAATGTAAC			
MS3	F1	CAAGCACTGTGGTTACTGTT	55	537	13
	R1	AAGTTAGGGCATTTAATAAAATTA			
	F2	GTTCAAGTAATTGATACCAGTCT	55		
	R2	CTCATTGAATCTAAATGTGTATAA			
MS4	F1	GCATATCGTCTCATAGGAACA	55	885	13
	R1	GTTCATGGTTATTAATTCCAGAA			
	F2	CGAAGTGTACTACATGTCTCT	55		
	R2	GGACTTTAATAAGTTACCTATAGT			
MS7	F1	GTTGATCGTCCAGATGGAATT	55	471	13
	R1	GACTATCAGTATTACTGATTATAT			
	F2	CAATAGTAAAGGAAGATGGTCA	55		
	R2	CGTCGCTTTGTTTCATAATCTT			

### Sequencing and phylogenetic analyses

Secondary PCR products were sequenced by Genscript Company (Nanjing, China). Sequences were aligned with reference sequences of *E. bieneusi* available in GenBank using the computer program Clustal X 1.83 and BLAST (http://www.ncbi.nlm.nih.gov/BLAST/). MEGA v5 (http://www.megasoftware.net/) was used to infer phylogenetic trees by the neighbor-joining (NJ) method (Kimura 2-parameter model). Bootstrapping (1000 replicates) was performed [28]. The *E. bieneusi* ITS genotypes were named according to the established nomenclature system [12].

### Statistical analysis

The variation in *E. bieneusi* prevalence (*у*) of foxes of different geographical location (*x*1), gender (*x*2), age (*x*3) and farming mode (*x*4) was analyzed using theχ2 test in SAS version 9.1 (SAS Institute Inc., USA). In a multivariable regression analysis, each of these variables was included in a binary Logit model as an independent variable. The best model was judged by Fisher’s scoring algorithm. All tests were two-sided, and results were considered statistically significant at *P* < 0.05. Odds ratios (ORs) and their 95 % confidence intervals (95 % CIs) were estimated to explore the strength of the association between *E. bieneusi*-positivity and each variable.

### Nucleotide sequence accession numbers

Representative nucleotide sequences have been deposited into GenBank with the following accession numbers: KT750157 to KT750167 for the ITS region, and KT844918 to KT844931 for the microsatellite loci (MS1, MS3, MS7).

## Results

### Prevalence of *E. bieneusi* in foxes

Of 302 fecal samples, 37 (12.25 %) were found to be *E. bieneusi*-positive by nested PCR amplification of the ITS region (Table 2). *Enterocytozoon bieneusi* was detected in each of the eight farms surveyed, and the highest infection rate was found in farm 8 (28.21 %, 11/39) (Table 3). There was no statistically significant difference in prevalence between males and females (*P* = 0.72) (Table 2). Adult foxes had a higher *E. bieneusi* prevalence compared with young foxes and pre-weaning foxes, but the difference was not significant (*P* = 0.39) (Table 2). Statistically significant differences were observed among provinces (Table 2). Foxes raised outdoors (26.03 % positive, 95 % CI 18.91–33.15) showed a significantly higher *E. bieneusi* prevalence compared to those raised indoors (5.77 % positive, 95 % CI 2.11–9.43, *P* < 0.0001) (Table 2).

**Table 2:** Factors associated with prevalence of *Enterocytozoon bieneusi* in farmed foxes in northern China

Factor	Category	No. tested	No. positive	% (95 % CI)	OR (95 % CI)	*P*-value
Region	Jilin Province	91	6	6.10 (0.92–11.28)	Reference	0.02
	Heilongjiang Province	70	6	8.57 (2.01–15.13)	1.33 (0.41–4.31)	
	Hebei Province	141	25	17.73 (11.43–24.04)	3.05 (1.20–7.77)	
Farming mode	Indoor	156	9	5.77 (2.11–9.43)	Reference	<0.0001
	Outdoor	146	38	26.03 (18.91–33.15)	5.75 (2.67–12.39)	
Gender	Male	139	16	11.51 (6.21–16.82)	Reference	0.72
	Female	163	21	12.88 (7.74–18.03)	1.14 (0.57–2.28)	
Age	Pre-weaned	64	6	9.38 (2.23–16.52)	Reference	0.39
	Young	180	21	11.67 (6.98–16.36)	1.28 (0.49–3.32)	
	Adult	58	10	17.24 (7.52–26.96)	2.01 (0.68–5.94)	
Total		302	37	12.25 (8.55–15.95)

**Table 3:** *Enterocytozoon bieneusi* genotypes identified in farmed foxes from different farms in northern China

Region	Farm ID	Age category (n)	No. positive/no. tested (%)	Genotype (*n*)
Jilin Province	1	Adults (2), Young (11), Pre-weaned (7)	2/20 (10 %)	NCF2 (*n* = 2)
	2	Adults (8), Young (19), Pre-weaned (9)	3/36 (8.33 %)	Peru8 (*n* = 1), Type IV (*n* = 1), D (*n* = 1)
	3	Adults (1), Young (12), Pre-weaned (22)	2/35 (5.71 %)	Type IV (*n* = 1), D (*n* = 1)
Heilongjiang Province	4	Adults (7), Young (17), Pre-weaned (2)	4/26 (15.38 %)	Peru8 (*n* = 1), Type IV (*n* = 1), CHN-DC1 (*n* = 1), NCF2 (*n* = 1)
	5	Adults (9), Young (27), Pre-weaned (7)	1/44 (2.27 %)	CHN-DC1 (*n* = 1)
Hebei Province	6	Adults (9), Young (34), Pre-weaned (2)	9/45 (20.00 %)	Peru8 (*n* = 2), NCF5 (*n* = 2), Type IV (*n* = 2), NCF1 (*n* = 2), NCF2 (*n* = 1)
	7	Adults (15), Young (33), Pre-weaned (9)	5/57 (8.77 %)	NCF2 (*n* = 4), NCF3 (*n* = 1)
	8	Adults (7), Young (26), Pre-weaned (6)	11/39 (28.21 %)	D (*n* = 2), NCF1 (*n* = 1), NCF2 (*n* = 5), NCF4 (*n* = 1), NCF6 (*n* = 1), NCF7 (*n* = 1)
Total		Adults (68), Young (178), Pre-weaned (56)	37/302 (12.25 %)	Peru8 (*n* = 4), Type IV (*n* = 5), D (*n* = 4), CHN-DC1 (*n* = 2), NCF1 (*n* = 3), NCF2 (*n* = 13), NCF3 (*n* = 1), NCF4 (*n* = 1), NCF5 (*n* = 2), NCF6 (*n* = 1), NCF7 (*n* = 1)

### Association between *E. bieneusi* positivity and exposure

The univariate analysis summarized in Table 2 shows a strong relationship between farming mode and *E. bieneusi*-positivity. The impacts of multiple variables on *E. bieneusi* were evaluated by a forward stepwise logistic regression analysis using Fisher’s scoring technique. In the final model, only one variable (farming mode) had an effect on prevalence, described by the equation y = 3.8786 –1.1977x4. Farming mode has a strong effect on the risk of *E. bieneusi* (Table 2).

### Genetic characterizations and genotype distribution of *E. bieneusi* in foxes

Analysis of the ITS region of *E. bieneusi* revealed four known genotypes, namely Peru 8, Type IV, CHN-DC1 and D [15, 24, 29]. In addition, seven novel genotypes (NCF1-NCF7) were detected (Table 3). A comparative analysis of the ITS sequences showed that the four known genotypes sequences of the identified *E. bieneusi* isolates were identical to that of Peru 8 (GenBank accession no. KJ668721), Type IV (accession no. KJ651436), CHN-DC1 (accession no. KJ710333) and D (accession no. JF776168) sequences available in GenBank, respectively.

Of the novel genotypes, NCF2 (*n* = 13, 35.14 %) was the most common, being found in 5 farms (farm 1 from Jilin province, farm 4 from Heilongjiang province, farms 6, 7 and 8 from Hebei province). The remaining novel genotypes were only present in Hebei province (Table 3).

### Phylogenetic relationships of *E. bieneusi* ITS genotypes

**Fig. 1:**
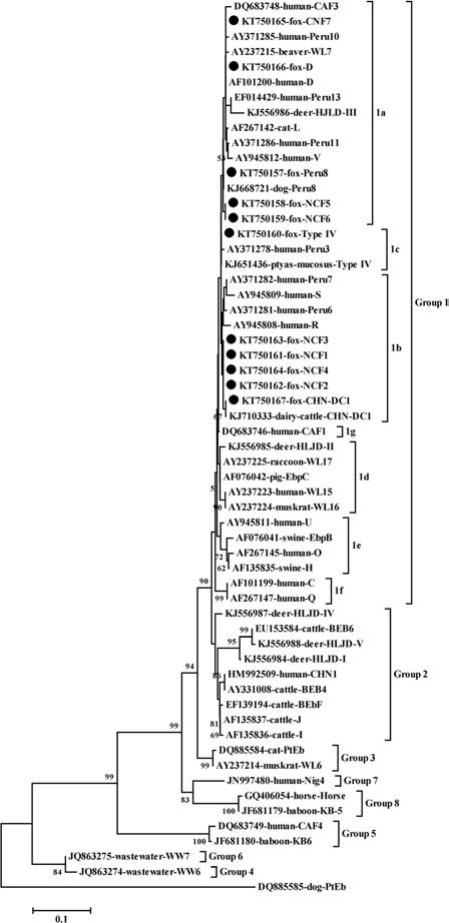
Phylogenetic analyses of *Enterocytozoon bieneusi* based on sequences of the internal transcribed spacer (ITS) region of the ribosomal RNA (rRNA) gene. The numbers at notes indicate bootstrap values. Bootstrap values below 50 % are not shown. Isolates identified in this study are indicated by solid circles

Phylogenetic analysis indicated that all the genotypes identified in this study belonged to group 1 (Fig. 1). NCF5 (*n* = 2), NCF6 (*n* = 1), NCF7 (*n* = 1), Peru8 (*n* = 4) and D (*n* = 4) were sub-classified into group 1a, with NCF5 and NCF6 forming a new clade; Type IV (*n* = 5) was in group 1c; NCF1 (*n* = 3), NCF2 (*n* = 13), NCF3 (*n* = 1), NCF4 (*n* = 1) and CHN-DC1 (*n* = 2) were grouped into group 1b, with NCF1NCF4 forming a new clade (Fig. 1).

### Multilocus genotyping

Three microsatellites (MS1, MS3 and MS7) loci and one minisatellite (MS4) locus were used to characterize the ITS-positive specimens. Twenty-one, 15, 0 and 17 *E. bieneusi* isolates were successfully amplified at MS1, MS3, MS4, and MS7, respectively. Sequence analysis revealed 7, 2, 0, and 5 genotypes at loci MS1, MS3, MS4 and MS7, respectively. Ten *E. bieneusi* isolates were successfully amplified at all three microsatellites (MS1, MS3 and MS7) (Table 4).

**Table 4:** Multilocus characterization of *Enterocytozoon bieneusi* isolates from farmed foxes in northern China

Code number	ITS genotype	Multilocus genotypes				
		MS1	MS3	MS4	MS7	GenBank Accession Nos.
189	NCF1	FVIII	FVI	-	FXI	KT844921, KT844925, −, KT844931
210	NCF1	FXI	FV	-	FX	KT844924, KT844926, −, KT844929
247	NCF2	FX	FVI	-	FXI	KT844923, KT844925, −, KT844931
251	NCF2	FVIII	FVI	-	FX	KT844921, KT844925, −, KT844929
272	NCF2	FXI	FV	-	FVIII	KT844924, KT844926, −, KT844930
280	NCF2	FXI	FV	-	FX	KT844924, KT844926, −, KT844929
200	Type IV	FXI	FVI	-	FX	KT844924, KT844925, −, KT844929
89	D	FVII	FVI	-	FX	KT844918, KT844925, −, KT844929
276	D	FIX	FV	-	FX	KT844922, KT844926, −, KT844929
199	Peru8	FXII	FVI	-	FVIII	KT844919, KT844925, −, KT844930

## Discussion

In the present study, 37 (12.25 %, 95 % CI 8.55–15.95) out of 302 farmed foxes were *E. bieneusi*-positive by the nested PCR amplification of the ITS rDNA. This prevalence was lower than that in farmed foxes (27.7 %, 53/191) in Harbin City, China [27], and also lower than that in wild foxes in the Spain (14.3 %, 1/7) [29] and USA (13.4 %, 9/67) [30]. The different prevalence may be due to different geo-ecological conditions, sample collection time, animal husbandry practices, animal welfare, age distribution of the samples, as well as sample sizes.

Ingestion of contaminated water and food is the most important means of transmission of *E. bieneusi* [9]. Therefore, density of foxes in a farm and environmental sanitation may strongly affect on prevalence. Stocking density of foxes is generally higher in farms in Hebei and Heilongjiang provinces relative to those in Jilin province. This may explain why farms from Hebei and Heilongjiang had higher *E. bieneusi* prevalences than those from Jilin province. Farming mode is another risk factor associated with *E. bieneusi* infection in farmed foxes. Foxes raised outdoors had a significantly higher prevalence than those kept indoors, probably due to the fact that outdoor foxes have more opportunities to make contact with contaminated environments and animals than indoor foxes.

Four *E. bieneusi* ITS genotypes, namely D, EbpC, WL11, and WL15, have been detected in foxes in the world [27, 29, 30]. Only one of these, D, was among the four known genotypes that we found in farmed foxes. We also found genotypes Peru8, CHN-DC1 and Type IV, and an additional seven novel genotypes (NCF1-NCF7). Thus, eleven *E. bieneusi* genotypes were endemic in farmed foxes in northern China. All these genotypes belonged to group 1 analyzed by NJ methods (Fig. 1), which suggests that farmed foxes are potential sources of human microsporidiosis. NCF2 was the most frequent genotype found in the present study. In contrast, only genotype D was found in farmed foxes in Harbin City in a previous study [27] and in wild foxes in Spain [29], and genotypes WL13 and WL15 were the most common in wild foxes in the USA [30].

All of the four known genotypes, CHN-DC1, D, Type IV and Peru8, have been previously found in northern China [15, 24, 31]. For example, genotype D was found in golden takins (*Budorcas taxicolor bedfordi*) in Shannxi [13], HIV patients and non-human primates in Henan [11, 21], dairy cattle, sheep, goats, pig, cats and dogs in Heilongjiang [24, 31–33]. Type IV was present in HIV patients and non-human primates in Henan [21], cats and dogs in Heilongjiang [15]. Peru8 was found in non-human primates in Henan [11], dogs in Heilongjiang [15]. CHN-DC1 was identified only in dairy cattle in Heilongjiang [31]. These findings suggest cross-transmission of *E. bieneusi* between foxes, other animal species, and humans. More importantly, genotypes D and Peru8 have also been found in drinking water in China, which suggests that we should pay more attention to this mode of transmission of *E. bieneusi*.

Recently, multilocus sequence typing (MLST) has been used to further study the taxonomy and population genetics of *E. bieneusi* using MS1, MS3, MS4 and MS7 [13, 15, 34]. In the present study, amplification of at least one locus was successful for every isolate. Amplification of MS4 was unsuccessful in every case. All the three microsatellite loci (MS1, MS3 and MS7) were only successfully amplified from ten isolates, revealing the presence of much diversity. These findings demonstrate the genetic diversity of *E. bieneusi* in farmed foxes.

## Conclusions

The present study revealed the existence (12.25 %, 37/302) of zoonotic *E. bieneusi* infection in farmed foxes in Jilin, Heilongjiang and Hebei provinces, northern China. Foxes raised outdoors displayed a significantly higher *E. bieneusi* prevalence than those raised indoors. This study also found ITS genotypes D, Peru8, CHN-DC1 and Type IV, and seven novel genotypes (NCF1-NCF7) in farmed foxes, and further diversity was revealed at three microsatellite loci combining with microsatellite locus for the first time. These results suggest that control strategies are required to limit *E. bieneusi* infection in farmed foxes, and to prevent transmission to humans and other animals.
